# Transcription Factor ANAC074 Binds to NRS1, NRS2, or MybSt1 Element in Addition to the NACRS to Regulate Gene Expression

**DOI:** 10.3390/ijms19103271

**Published:** 2018-10-21

**Authors:** Lin He, Jingyu Xu, Yucheng Wang, Kejun Yang

**Affiliations:** 1 Key Laboratory of Modern Agricultural Cultivation and Crop Germplasm Improvement of Heilongjiang Province, College of Agriculture, Heilongjiang Bayi Agricultural University, 5 Xinfeng Road, Daqing 163319, China; linlinhe65@sina.com (L.H.); xujingyu2003@hotmail.com (J.X.); 2 State Key Laboratory of Tree Genetics and Breeding, Northeast Forestry University, 26 Hexing Road, Harbin 150040, China

**Keywords:** *cis*-acting element, gene expression regulation, protein–DNA interaction, NAC, yeast one-hybrid

## Abstract

NAC (NAM, ATAF1/2, and CUC2) transcription factors play important roles in many biological processes, and mainly bind to the NACRS with core sequences “CACG” or “CATGTG” to regulate gene expression. However, whether NAC proteins can bind to other motifs without these core sequences remains unknown. In this study, we employed a Transcription Factor-Centered Yeast one Hybrid (TF-Centered Y1H) screen to study the motifs recognized by ANAC074. In addition to the NACRS core cis-element, we identified that ANAC074 could bind to MybSt1, NRS1, and NRS2. Y1H and GUS assays showed that ANAC074 could bind the promoters of ethylene responsive genes and stress responsive genes via the NRS1, NRS2, or MybSt1 element. ChIP study further confirmed that the bindings of ANAC074 to MybSt1, NRS1, and NRS2 actually occurred in *Arabidopsis*. Furthermore, ten NAC proteins from different NAC subfamilies in *Arabidopsis thaliana* were selected and confirmed to bind to the MybSt1, NRS1, and NRS2 motifs, indicating that they are recognized commonly by NACs. These findings will help us to further reveal the functions of NAC proteins.

## 1. Introduction

Transcription factors (TFs) are a group of regulatory proteins which can regulate the expression of target genes through binding to specific cis-regulatory elements that are often located in the promoters of target genes [1]. Although the genes encoding the TFs only account for a small portion in plant genome, they still play pivotal roles in the intricate regulated networks [2].

NAC (NAM, ATAF1/2, and CUC2) proteins are plant-specific transcription factors and constitute a large family with 117 members in *Arabidopsis* [3]. NAC TFs contain a highly conserved NAC domain located at the N-terminal region, which is responsible for DNA-binding activity or dimerization, and a diversified domain at C-terminal, which determines transcription activity [4]. Emerging research indicates that NAC TFs regulate a diverse range of processes in plants, such as embryo and shoot meristem development [5], root development [6], xylem development [7], leaf senescence [8,9], formation of secondary walls [10], and hormone signaling [11,12]. In addition, NAC TFs have also been shown to participate in the regulation of stress responses including salt [13], cold [14,15], drought [13,16,17], and fungal infection [18].

The diverse functions of NAC TFs possibly depends on their combination with the relevant cis-acting elements existing in the promoters of their target genes. Therefore, target element identification for NAC TFs is a prerequisite for parsing the action of NAC family members in the plant. Accumulated studies have shown that NAC transcription factors mainly bind to the classic NAC recognition sequence (NACRS) with discontinuous core sequence of CATGTG and CACG in promoters to regulate the expression of target genes. In *Arabidopsis*, ANAC019, ANAC055, and ANAC072 bind to a NACRS in the promoter of the early responsive to dehydration stress 1 (*ERD1*) gene, which encodes a ClpA (ATP binding subunit of the caseinolytic ATP-dependent protease) homologous protein [19]. ANAC096 activates transcription via recognition of the NACRS sequences within the *RD29A* promoter [20]. SNAC1 from rice can bind to the similar NACRS in the promoter of the rice *ERD1* (early responsive to drought 1) gene [13]. In addition to discontinuous motif, NACs also bind to the continuous core sequence. For instance, both ANAC019 and ANAC092 from *Arabidopsis* can bind to the TTNCGT[G/A] sequence [21]. *Arabidopsis* NAC1 binds to a 21-bp DNA fragment containing an as-1 element (TGACG) [6]. ANAC069 binds to C[A/G]CG[T/G] sequence to regulate the expression of stress related genes under salt and osmotic stress conditions [22]. Combining these results, it can be suggested that diverse NAC proteins can bind cis-acting elements with similar core sequences “CACG” or “CGT[G/A]”.

To date, although the functions of NACs have been studied extensively, there are still many grey areas that need to be further studied in the NAC-mediated gene regulatory networks. There has been little work on new target element identification for NAC TFs. Therefore, we were convinced that it was worthwhile pursuing the challenge to identify more target elements of NACs.

In the present study, we identified three new interactions between NACs and DNA sequences in *Arabidopsis* using the TF-centered Y1H system [23,24], including two novel motifs, NRS1 and NRS2, and an MybSt1 that previously was found to only bind by Myb transcription factors. Further analysis showed that ANAC074 could bind the promoters of ethylene and stress responsive genes via the NRS1, NRS2, or MybSt1 element. These results suggested that NACs recognize more motifs than we previously knew and these motifs may play roles in expression regulation mediated by NACs. The identified motifs recognized by NACs will facilitate understanding of the function of NACs in depth.

## 2. Results and Discussion

### 2.1. Identification of the DNA Motifs Bound by ANAC074

The TF-Centered Y1H system is a simple and efficient method for identification of the DNA sequence bound by transcription factors of interest [23,24]. To identify the DNA sequences recognized by ANAC074, the random DNA insertion prey library was screened with ANAC074 (AT4G28530) cloned into pGADT7-Rec2 as bait using the Y1H system [23,24]. Eight positive clones were obtained. After further selection, four positive clones were identified to have high binding affinity to ANAC074, and were sequenced. Since the two flanking sequences of the insertion sequence may also be part of cis-acting elements recognized by ANAC074, three bases of each flanking sequence together with the insertion sequence were used for further analysis. The insertion sequences were firstly predicted with PLACE and PlantCare to find the putative motifs bound by ANAC074. The insertion sequences and the predicted motifs are shown as Table 1.

The sequence “CCCCTTCACGCGGG” (insertion is underlined) contains the core sequence “CACG”. Previous studies have shown that NAC transcription factors mainly bind to the classic NACRS with discontinuous core sequence of CATGTG and CACG in promoters to regulate the expression of target genes [13,19,20]. In addition to discontinuous motif, some NACs can also bind continuous core sequence. *Arabidopsis* ANAC019 and ANAC092 could bind to continuous TTNCGT[G/A] sequence [21], the conserved sequence “CGT[G/A]” is similar to the core sequence “CACG” (its reverse complementary sequence CGTG) of NACRS. *Arabidopsis* ANAC069 protein was found to bind to C[A/G]CG[T/G] sequence, in which the sequence C[A/G]CG is similar to the core sequence “CACG” [22]. *Arabidopsis* NAC1 binds to a 21-bp DNA fragment containing an as-1 element (TGACG), in which the conserved sequence “GACG” is also similar to the “CACG” [6]. In this examination, using ANAC074 as bait, one of the cis-acting elements we caught was “CCCCTTCACGCGGG” containing the core sequence “CACG” like other NAC binding elements. This result further suggested that some NAC proteins can bind core sequences “CACG”.

However, other NAC proteins appear to bind different sequences. Kim et al. (2007) found that the *Arabidopsis* transcriptional repressor CBNAC can bind to CBNACBS with a “GCTT” sequence but not the “CACG” sequence [25]. Therefore, we further studied the other three insertion sequences without “CACG”. According to PLACE and PlantCare prediction, the sequence “CCCGGATAACGGGG” includes MybSt1-binding element “GGATA” [26], “CCCTGAATCTCGGG” includes ARR1AT motif “NGATT”, and “CCCTTAGTTTCGGG” contains LTRE-1 motif “CCGAAA” (Table 1). These results implied that the ANAC074 may bind to MybSt1-binding element (shown as MybSt1 in the following), ARR1AT and LTRE-1 motifs. To verify this hypothesis, three tandem copies of “GGATA”, “TGATT”, and “CCGAAA” were respectively cloned into pHIS2. The bindings of ANAC074 to these three sequences were investigated by Y1H assay. The results showed that the ANAC074 can bind to “GGATA”, but cannot bind to the sequence of “TGATT” and “CCGAAA”, indicating that ANAC074 binds to MybSt1 element, but cannot interact with ARR1AT and LTRE-1 motif. Therefore, the sequence of “CCCTTAGTTTCGGG” and “CCCTGAATCTCGGG” both contain unknown DNA motifs recognized by ANAC074, and were further studied.

### 2.2. Analysis of the Unknown Elements Recognized by ANAC074

**Figure 1:**
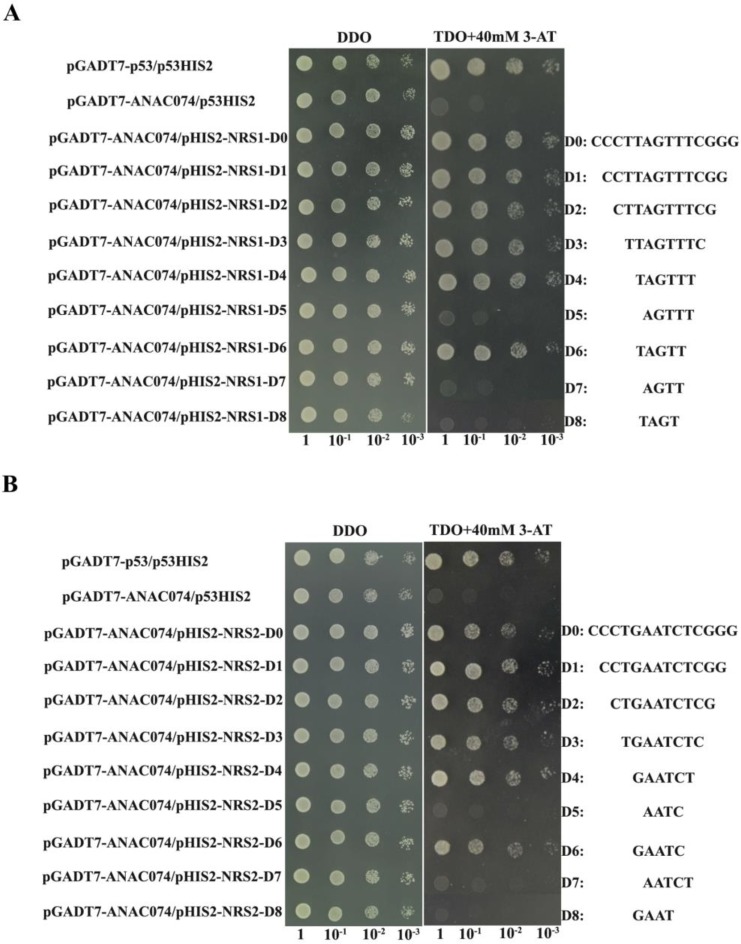
Serial deletion of the inserted sequence to determine the motif recognized by ANAC074. (**A**) Serial deletion of the inserted sequence to determine the exact sequence of NRS1 recognized by ANAC074. (**B**) Serial deletion of the inserted sequence to determine the exact sequence of NRS2 recognized by ANAC074. pGADT7-ANAC074: pGADT7-Rec2 vector that encodes ANAC074 fused with GAL4 AD as the effector construct. pHIS2-NRS1-D0-8/NRS2-D0-8: Three tandem copies of sequence of D0 to D8 of NRS1/NRS2 were respectively cloned into the pHIS2 to drive the *HIS3* reporter gene as the reporter constructs. The right panel shows the sequence of different NRS1 or NRS2 deletions. Positive control: pGADT7-p53 interacting with p53HIS2. Negative control: pGADT7-ANAC074 interacting with p53HIS2. Positive transformants were further confirmed by spotting yeast cells with serial dilutions (1/1, 1/10, 1/100, 1/1000) onto medium of SD/-His/-Leu/-Trp with 40 mM 3-AT. The transformants grown at SD/-Leu/-Trp were used as positive controls for transformant growth.

To determine the borders of the two unknown elements, we serially deleted the sequences of “CCCTTAGTTTCGGG” and “CCCTGAATCTCGGG” (insertion is underlined) from both 5’ and 3’ terminals, and cloned the sequences into pHIS2 to drive the *HIS3* gene. The interaction between these deleted insertion sequences and ANAC074 was studied using Y1H. The sequences of “AGTT” and “TAGT” abolished the binding to ANAC074 while the sequence of “TAGTT” restored the binding activity (Figure 1A). This result suggested that the DNA sequence recognized by ANAC074 was “TAGTT” (named NRS1). Similarly, the sequences of “AATC” and “GAAT” disrupted binding to ANAC074 while the sequence of “GAATC” restored the binding activity (Figure 1B). Therefore, the sequences recognized by ANAC074 were “GAATC” (named NRS2).

### 2.3. Binding of Two Novel Motifs and MybSt1 Motif to ANAC074

**Figure 2:**
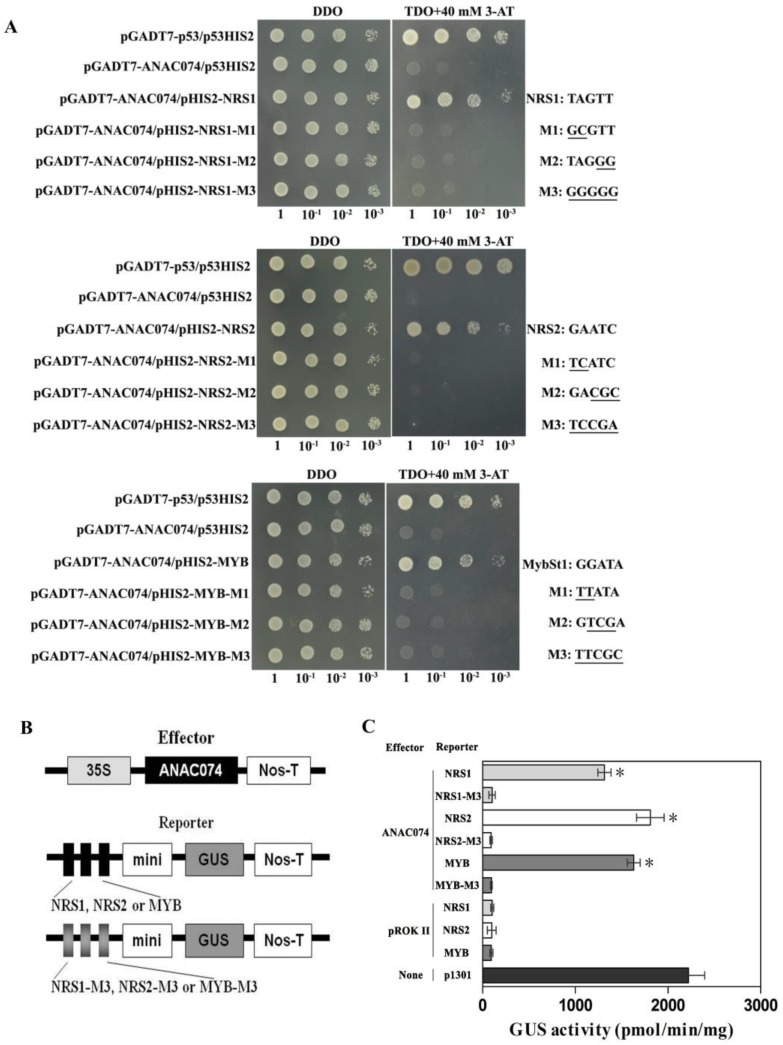
The bindings of NRS1, NRS2 and MybSt1 to ANAC074. (**A**) Analysis of the specific bindings of NRS1, NRS2, and MybSt1 to ANAC074 using Y1H assay. The right panel showed the sequences of NRS1, NRS2, MybSt1 motifs and their respective mutants (M1, M2 and M3). Positive transformants were confirmed by spotting yeast cells with serial dilutions (1/1, 1/10, 1/100, 1/1000) onto medium of SD/-His/-Leu/-Trp with 40 mM 3-AT. Positive control: pGADT7-p53 interacting with p53HIS2; Negative control: pGADT7-ANAC074 interacting with p53HIS2. The transformants grown at SD/-Leu/-Trp were used as positive controls for transformant growth. (**B**) Schematic diagram of the reporter and effector vectors. The effector was constructed by cloning the ORF of ANAC074 into pROKII under 35SCAMV promoter. Three tandem copies of NRS1, NRS2, and MybSt1 (black shading) or their complete mutant types NRS1-M3, NRS2-M3, and MYB-M3 (grey shading) were respectively fused to the minimal 35S promoter for driving *GUS* as reporter vectors. (**C**) Transient co-transformation of effector and reporter constructs into tobacco leaves to study the specific bindings of NRS1, NRS2, and MybSt1 to ANAC074. GUS activity was determined to study the binding affinity between the test DNA sequence and ANAC074, and the combinations between effector and reporter construct are shown in the left panel. The empty pCAMBIA1301 was as positive control. Co-transformed empty pROKII with pCAM-NRS1, pCAM-NRS2 or pCAM-MYB were as negative controls. Data represent mean values of three independent experiments, and bars indicate standard deviation (SD). Asterisks indicate a significant difference compared with the mutation (Student’s test; * *p* <0.05).

The bindings of ANAC074 to NRS1 (TAGTT), NRS2 (GAATC), MybSt1 (GGATA) and their mutants were investigated by Y1H to investigate the specification of binding. The results showed that the ANAC074 can bind to MybSt1 element, NRS1 and NRS2, while the MybSt1 mutants (MYB-M1: TTATA; MYB-M2: GTCGA; MYB-M3: TTCGC), NRS1 mutants (NRS1-M1: GCGTT, NRS1-M2: TAGGG; NRS1-M3: GGGGG), and NRS2 mutants (NRS2-M1: TCATC; NRS2-M2: GACGC; NRS2-M3: TCCGA) all lost binding activity with ANAC074, indicating that the bindings of ANAC074 to MybSt1 element, NRS1 and NRS2 are specific (Figure 2A). Furthermore, transient coexpression of ANAC074 and MybSt1 element, NRS1, and NRS2 or their complete mutants (NRS1-M3: GGGGG; NRS2-M3: TCCGA; MYB-M3: TTCGC) in tobacco also confirmed that the ANAC074 bound to MybSt1, NRS1 and NRS2 elements specifically (Figure 2B,C). Although various cis-elements for NAC proteins have been identified [6,13,19,20,21,22,25], the NRS1, NRS2, and MybSt1 binding sequences for ANAC074 are different from those reported previously. These results suggested different NAC transcription factors may bind to different core element sequences.

The transcription factors regulate gene expression by binding to certain motifs. Therefore, their function could be predicted by their binding motifs. ANAC074 protein can bind to other cis-acting elements, in addition to sequences containing “CACG” or its complement, suggesting that the regulatory function of ANAC074 is different from that of other NAC domain proteins like ANAC019, ANAC055, and ANAC072.

Furthermore, the MybSt1 motif was found previously to be bound by MYB transcription factors MybStl that encode a novel Myb-related protein from potato [26]. MybStl requires a GGATA-containing sequence for high-affinity binding. GGATG possesses a 20–30 times lower binding affinity than GGATA, which suggested the fifth “A” in the GGATA sequences is important for MybStl recognition [26]. Our results showed that ANAC074 could recognize the GGATA (MybStl), but failed to interact with the mutated motifs TTATA (M1), GTCGA (M2) and TTCGC (M3) (Figure 2A), indicating that the bases “GG” and “GAT” are also essential to ANAC074 to bind to MybSt1 motif. MYB transcription factors perform diverse critical functions in plants, including secondary metabolism [27], development [28], hormonal signaling [29], and biotic stress response [30]. Increasing evidence in model plants show that MYB genes are also involved in tolerance to abiotic stress [31,32]. The binding of ANAC074 to the MybSt1 motif suggested that it might have similar functions to the MYB TFs. NAC proteins may share regulatory networks with MYB proteins by binding to the MybSt1 binding site. The two TFs can be competitive or cooperative in the same signaling pathway.

### 2.4. ANAC074 Can Bind the Promoters of AT4G17500, AT1G74930 and AT4G11280 via the NRS1, NRS2 or MybSt1 Element

The MybSt1, NRS1, and NRS2 were identified using the TF-centered Y1H system [23,24]. However, to eliminate artifacts, we further searched the NRS1, NRS2, and MybSt1 sequences to find the genes that were putatively regulated by ANAC074 from *Arabidopsis* using the Patmatch program on Tair [33]. The criteria for promoter selection were the promoter regions (0 to −1500 bp) of genes containing fewer kinds of the motifs of MybSt1, NRS1, and NRS2, but have more copies of the contained motif. We found a total of 33 genes that met the criteria. Three of them (*AT4G17500*, *AT1G74930* and *AT4G11280*) happen to be the target genes of ANAC069 [22]. We speculated that these three genes are probably also the target genes of ANAC074. Therefore, these three genes were selected for the promoter mutagenesis.

**Figure 3:**
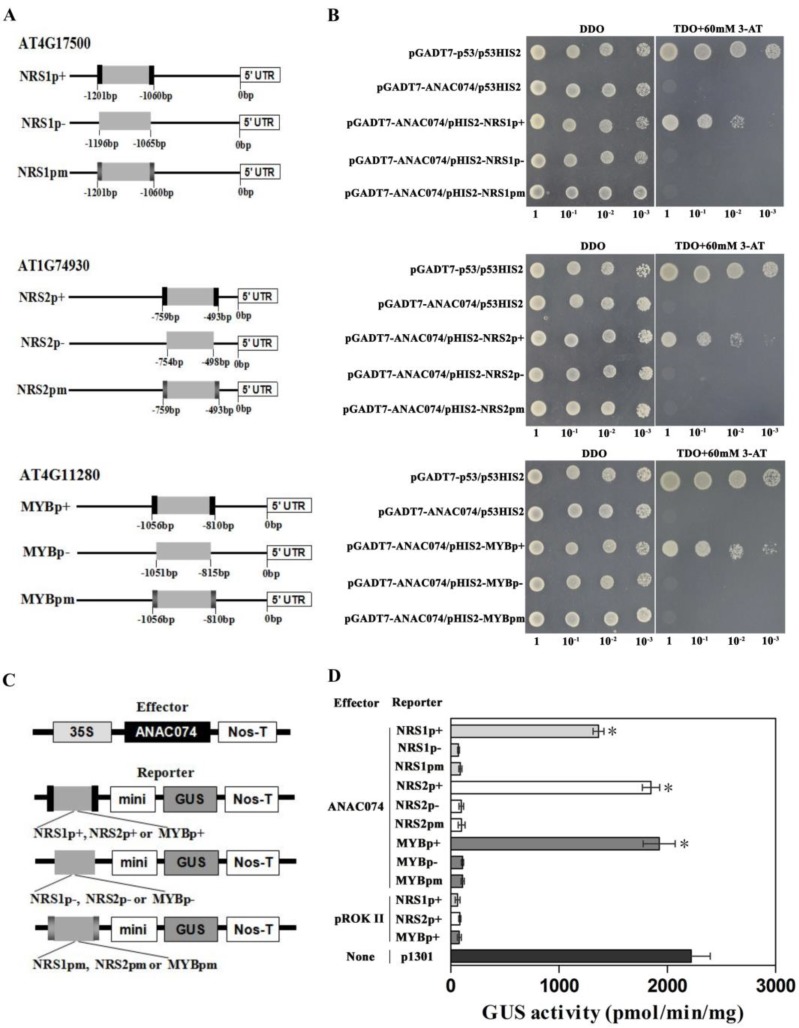
ANAC074 can bind the promoter of *AT4G17500*, *AT1G74930,* and *AT4G11280* via the NRS1, NRS2 or MybSt1 element. (**A**) Schematic diagram of the truncated promoters containing NRS1, NRS2, or MybSt1, without NRS1, NRS2, or MybSt1, or with the mutated NRS1, NRS2, or MybSt1 used in Y1H analysis. NRS1p+, NRS2p+, MYBp+: the truncated promoter with NRS1, NRS2, or MybSt1. NRS1p-, NRS2p-, MYBp-: the truncated promoter without NRS1, NRS2, or MybSt1. NRS1pm, NRS2pm, MYBpm: the truncated promoter with the completely mutated NRS1, NRS2, or MybSt1. Black shading indicates NRS1, NRS2, or MybSt1 motif. Dark grey shading indicates completely mutated NRS1, NRS2, or MybSt1 motif. (**B**) Analyses of the binding of ANAC074 to the truncated promoters containing NRS1, NRS2, or MybSt1 motif using Y1H. Positive transformants were further identified by spotting yeast cells with serial dilutions (1/1, 1/10, 1/100, 1/1000) onto medium of SD/-His/-Leu/-Trp with 60 mM 3-AT. The transformants grown at SD/-Leu/-Trp were as positive controls for transformant growth. Positive control: pGADT7-p53 interacting with p53HIS2; Negative control: pGADT7-ANAC074 interacting with p53HIS2. (**C**) Schematic diagram of the effector and reporter constructs used in vivo transient expression assays. (**D**) Analyses of the bindings of ANAC074 to the truncated promoters containing NRS1, NRS2, or MybSt1 motif used in the coexpression experiments. The different combinations between effector and reporter are shown in the left panel. The empty pCAMBIA1301 was as positive control. Co-transformations of empty pROKII with pCAM-NRS1p+, pCAM-NRS2p+ or pCAM-MYBp+ were as negative controls. Data represent mean values of three independent experiments, and bars indicate standard deviation (SD). Asterisks indicate a significant difference compared with NRS1p-, NRS2p-, or MYBp- (Student’s test; * *p* < 0.05).

The promoter analysis showed that NRS1 was present in the promoter regions of *ATERF-1 (AT4G17500)*, NRS2 was present in the promoter regions of *ORA47 (AT1G74930)*, and MybSt1 element was included in *ATACS6 (AT4G11280)* (Figure 3A). To study whether ANAC074 can bind to the promoters containing MybSt1, NRS1, or NRS2 element, the truncated promoter of *AT4G17500* (no NACRS, NRS2 or MybSt1 element was included) containing “TAGTT” (NRS1p+), without “TAGTT” (NRS1p-) or with “GGGGG” (NRS1pm: completely mutated NRS1); truncated promoter of *AT1G74930* (no NACRS, NRS1 or MybSt1 element was included) containing “GAATC” (NRS2p+), without “GAATC” (NRS2p-) or with “TCCGA” (NRS2pm: completely mutated NRS2); truncated promoter of *AT4G11280* (no NACRS, NRS1 or NRS2 was included) containing “GGATA” (MYBp+), without “GGATA” (MYBp-) or with “TTCGC” (MYBpm: completely mutated MybSt1), were respectively inserted into pHIS2 and hybridized with ANAC074 using Y1H assay. The results showed that ANAC074 binds to the truncated promoters with NRS1, NRS2, or MybSt1, but failed to bind with the truncated promoters without or with completely mutated NRS1, NRS2, or MybSt1 (Figure 3B). Coexpression of effector and reporter vectors in tobacco also confirmed that GUS activity was detected when coexpression of *ANAC074* and *GUS* gene driven by the truncated promoters with NRS1, NRS2, or MybSt1. However, GUS activity was not detected when coexpression of *ANAC074* and *GUS* gene was driven by the truncated promoters without or with completely mutated NRS1, NRS2, or MybSt1 (Figure 3C,D). Taken together, these results suggested that the promoters with NRS1, NRS2, and MybSt1 motifs were recognized by ANAC074.

In the previous study, we found that *AT4G17500*, *AT1G74930,* and *AT4G11280* were target genes regulated by ANAC069 under salt stress condition using Afymetrix Arabidopsis Gene Chips (GSE82274). In this study, ANAC074 binds to the NRS1, NRS2, or MybSt1 motifs in the promoters of *AT4G17500*, *AT1G74930,* and *AT4G11280*. ANAC069 and ANAC074 share down-stream target genes, indicating that there are similar regulatory mechanisms between ANAC069 and ANAC074 and that their transactivation functions are somewhat redundant. The function of ANAC069 in salt and osmotic stress has been confirmed [22], and whether ANAC074 also plays a role in abiotic stress requires further studies.

### 2.5. Binding of ANAC074 to NRS1, NRS2 and MybSt1 Actually Occurs in Plants

**Figure 4:**
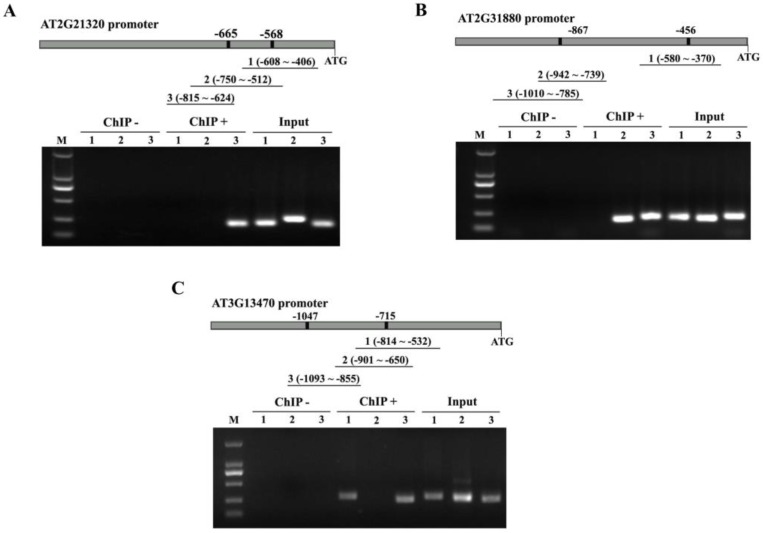
ChIP analysis of ANAC074 binding to NRS1, NRS2, and MybSt1. PCR products amplified from the promoters of *AT2G21320* (**A**), *AT2G31880* (**B**), and *AT3G13470* (**C**) were size fractionated by gel electrophoresis. The promoter of *AT2G21320* contains NRS1 motifs and without NRS2, Mybst1 and NACR motifs; the promoter of *AT2G31880* includes NRS2 and no NRS1 and MybSt1 and NACRS; and the promoter of *AT3G13470* contains only MybSt1 and no NRS1, NRS2, and NACRS. Black lines represent the promoter regions for PCR amplification, and their positions in promoters are also shown. Input, Input DNA was used as the control; CHIP+: chromatin immunoprecipitated with anti-GFP antibody and PCR amplified; CHIP-: chromatin immunoprecipitated with HA antibody and PCR amplified.

To determine whether the binding of ANAC074 to NRS1, NRS2 and MybSt1 actually occurs in plants, ChIP analysis was performed using *Arabidopsis thaliana* plants overexpressing a ANAC074-GFP fusion gene. The three truncated promoter regions of each putative target gene (*AT2G21320*, *AT2G31880*, *AT3G13470*, *AT4G17500*, *AT1G74930*, or *AT4G11280*) with NRS1, NRS2 or MybSt1 element were examined. The results showed that the truncated promoters of *AT2G21320*, *AT2G31880* and *AT3G13470* all can be amplified from chromatin immunoprecipitated with anti-GFP antibody, but not from chromatin immunoprecipitated with HA antibody (Figure 4). These results suggested that ANAC074 binds to the promoter of *AT2G21320* (containing NRS1 in promoter and no NRS2, MybSt1, and NACRS) (Figure 4A), the promoter of *AT2G31880* (containing NRS2 in promoter and no NRS1, MybSt1, and NACRS) (Figure 4B), and the promoter of *AT3G13470* (containing MybSt1 in promoter and no NRS1, NRS2, and NACRS) (Figure 4C). Unfortunately, we did not detect that the promoters of *AT4G17500*, *AT1G74930*, or *AT4G11280* could be bound by ANAC074 in the ChIP experiment. The truncated promoters of *AT4G17500*, *AT1G74930*, and *AT4G11280* could be amplified from input; however, the chromatin DNA of 35S::ANAC074::GFP transgenic plants immunoprecipitated with GFP antibody (ChIP+) failed to amplify the corresponding PCR products (data not shown).

Interestingly, there are regions where the binding site is present but no PCR product amplification is detected (Figure 4A–C). This phenomenon is also present in the study of Feng et al. [34]. They examined a total of six areas containing target components, and the results showed that MdCIbHLH1 only bound to the *MdCBF2* promoter in three regions [34]. It is likely that different DNA fragments containing assumed binding sites compete when binding to the same transcriptional factor. Additionally, it is also possible that a certain amount of DNA was lost in the process of DNA purification after uncrosslinking.

Accumulating evidence indicates that NAC gene family members are involved in regulating ethylene signals [35,36]. MdNAC047 directly binds to the *MdERF3* (ETHYLENE RESPONSE FACTOR) promoter and activates its transcription. MdNAC047 regulates ethylene production at least partially in an MdERF3-dependent pathway [37]. SNAC4 and SNAC9 could positively regulate the tomato fruit ripening process by functioning upstream of ethylene synthesis genes including *LeACS2* and *LeACS4* [38]. It is worth mentioning that *ATERF-1* (*AT4G17500*), *ORA47* (*AT1G74930*), *ACS6* (*AT4G11280*), *BBX18* (*AT2G21320*), *SOBIR1* (*AT2G31880*), and *CPNB2* (*AT3G13470*) as well as *ANAC074* all can respond to ethylene (see Affymetrix Arabidopsis ATH1 Genome Array, GSE537), which suggests they may be involved in the ethylene signaling pathway.

ATERF-1 (AT4G17500) and ORA47 (AT1G74930) both belong to the ERF/AP2 transcription factor family, which plays a crucial role in the ethylene signaling pathway [39,40]. ATERF-1 encodes a member of the ERF (ethylene response factor) subfamily B-3, and ORA47 encodes a member of the DREB subfamily A-5. ACS6 is important to the SA potentiated induction of ethylene biosynthesis [41]. In Y1H and transient expression assays we found that ANAC074 can bind to the promoters of *ATERF-1*, *ORA47*, and *ACS6*, demonstrating that ANAC074 may be as a regulator functioning upstream of ethylene synthesis genes. However, we did not detect the interaction between ANAC074 and the promoters of these three genes using the ChIP experiment in 3-week-old seedlings under normal growth condition. On the one hand, there may be functional redundancy between ANAC074 and other NACs under normal growth conditions in *Arabidopsis*. It is possible that the promoters of *ATERF-1*, *ORA47*, and *ACS6* are preferentially identified by ANAC069 or other NACs rather than ANAC074 when *Arabidopsis* grows normally. On the other hand, the interaction between ANAC074 and the promoter of *ATERF-1*, *ORA47*, or *ACS6* in *Arabidopsis* may be determined by the growth period and the environmental conditions of the plants. Perhaps this interaction occurs only during specific growth stage or under certain adverse conditions. Further study was required to reveal when and under what conditions ANAC074 binding to the promoters of *ATERF-1*, *ORA47*, and *ACS6* occurs in *Arabidopsis thaliana*.

Some NAC transcriptional factors are involved in biotic or abiotic stress response through the ethylene signaling pathway. For example, *Arabidopsis* transcription factor NAC2 is involved in the salt stress response through the ethylene signaling pathway [36]. An et al. found a novel “MdNAC047-*MdERF3*-ethylene-salt tolerance” regulatory pathway, which provide new insight into the link between ethylene and salt stress [37]. In this work, ANAC074 can bind to the promoters of *BBX18* (*AT2G21320*), *SOBIR1* (*AT2G31880*) and *CPNB2* (*AT3G13470*) via NRS1, NRS2 or MybSt1 in *Arabidopsis*, respectively. The function of *BBX18* and *SOBIR1* in *Arabidopsis* response to stress has been confirmed. Heat stress responsive gene *BBX18* plays a negative role in thermotolerance [42]. Under-expression of *BBX18* displayed both increased basal and acquired thermotolerance in the transgenic plants, while over-expression of *BBX18* reduced tolerance to heat stress in transgenic lines [42]. At the same time, the expression of *ANAC074* was down-regulated gradually by heat (37 °C) treatment compared with the normal condition (22 °C) (see Affymetrix Arabidopsis ATH1 Genome Array, GSE4062), which suggested ANAC074, as an potential upstream regulator of *BBX18*, is likely to play an important role under heat stress. In addition, *SOBIR1* (*AT2G31880*) participates in regulating biotic stress response including *Magnaporthe oryzae* [43] and fungal infection [44]. The function of *SOBIR1* in biotic stress has been confirmed and whether *ANAC074* also plays a role in biotic stress requires further studies.

### 2.6. Comparison of the Binding Abilities of ANAC074 to NRS1, NRS2, MybSt1, and NACRS

**Figure 5:**
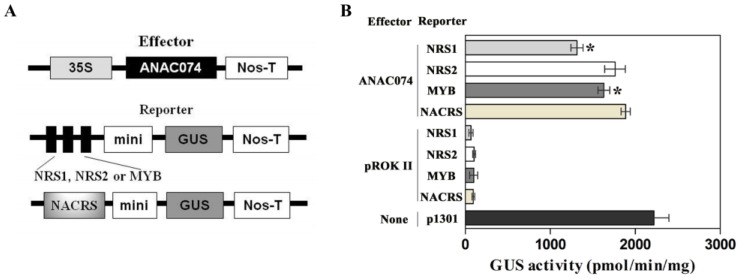
Comparison of the binding affinities of NACRS, NRS1, NRS2, and MybSt1 to ANAC074. (**A**) Schematic diagram of the reporter and effector constructs. (**B**) Three tandem copies of NRS1, NRS2, MybSt1, or one copy of NACRS were respectively fused with minimal promoter to drive *GUS*, and co-transformed with ANAC074 under control of 35S promoter into tobacco leaves. GUS activity was determined to study their binding affinity. The empty pCAMBIA1301 was used as positive control. Co-transformed empty pROKII with pCAM-NRS1, pCAM-NRS2, pCAM-MYB, or pCAM-NACRS were as negative controls. Data represent mean values of three independent experiments, and bars indicate standard deviation (SD). Asterisks indicate a significant difference compared with NACRS (Student’s test; * *p* < 0.05).

Since the NACRS motif, containing the “CACG” core sequence, has been identified to be recognized by a lot of NAC TFs [13,19,20]. Our results showed that NAC TFs can also bind to NRS1, NRS2, and MybSt1. Therefore, the binding abilities of ANAC074 to these cis-acting elements were further compared using transient expression assays in vivo. Schematic diagram of the effector and reporter constructs used in vivo transient expression assays is shown in Figure 5A. GUS activity analysis showed that ANAC074 displayed the lowest binding ability to NRS1, followed by MybSt1, NRS2, and NACRS (Figure 5B). But no remarkable differences existed in binding activity between NRS2 and NACRS (Figure 5B). These results demonstrated that ANC074 binds to NACRS, NRS1, NRS2, or MybSt1 with different affinities.

### 2.7. Binding of NRS1, NRS2, and MybSt1 to NAC (NAM, ATAF1/2, and CUC2) Family Members

**Figure 6:**
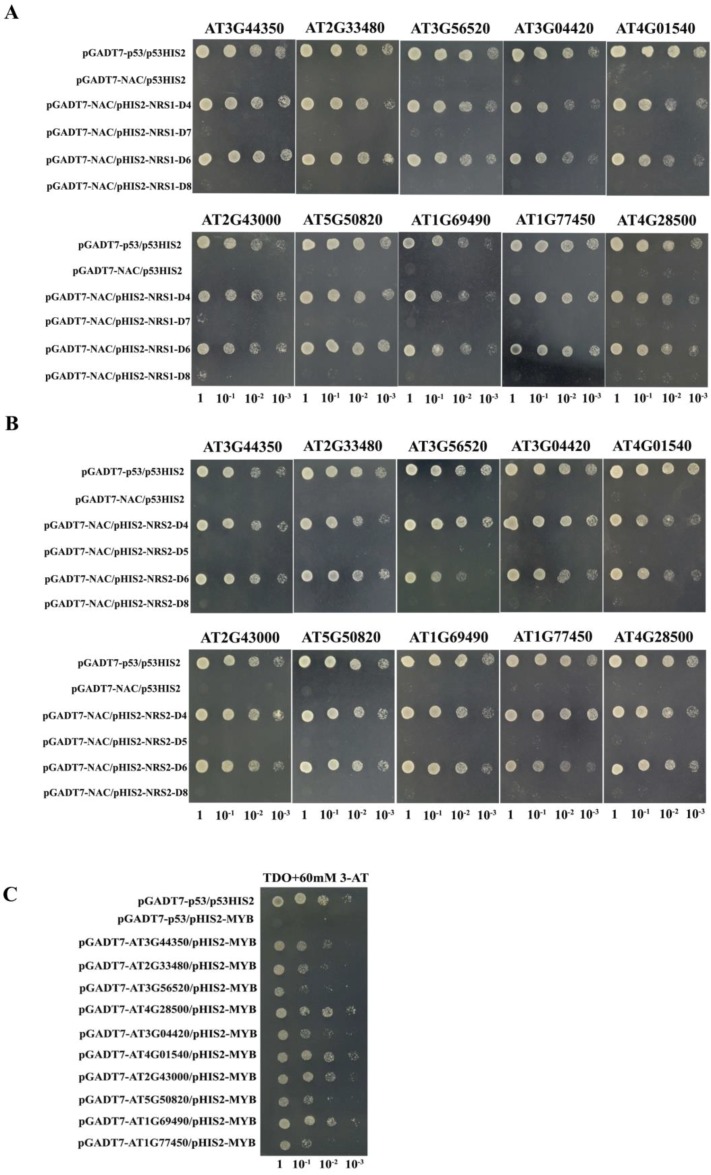
Bindings of NRS1, NRS2, and MybSt1 to other NACs from *Arabidopsis*. The bindings of ten NACs from different NAC subfamily to NRS1 (**A**), NRS2 (**B**), and MybSt1 (**C**). The ORFs of ten NACs were respectively fused with GAL4 AD in pGADT7-Rec2 as the effector constructs. pHIS2-NRS1-D4, -D7, -D6, -D8 or pHIS2-NRS2-D4, -D5, -D6, -D8: Three tandem copies of NRS1-D4, -D7, -D6, -D8, or NRS2-D4, -D5, -D6, -D8 (see Figure 1 for the sequence of these elements) were respectively cloned into the pHIS2 as the reporter constructs. Positive transformants were identified by spotting yeast cells with serial dilutions (1/1, 1/10, 1/100, 1/1000) onto medium of SD/-His/-Leu/-Trp with 60 mM 3-AT. Positive control: pGADT7-p53 interacting with p53HIS2; Negative control: pGADT7-ANAC074 interacting with p53HIS2.

Researchers identified 117 NAC genes in *Arabidopsis* [3]. On the basis of the phylogenetic analysis, the *Arabidopsis* NAC family was divided into ten major polytomies by collapsing branches with bootstraps <40% [45]. To study the binding of NRS1, NRS2, and MybSt1 to other NAC members, the NACs were selected from group III (AT2G33480/ANAC041, AT1G69490/ANAC029 and AT1G77450/ANAC032), group V (AT2G43000/ANAC042), group VI (AT3G44350/ANAC061 and AT5G50820/ANAC097), group VII (AT3G04420/ANAC048 and AT4G01540/ANAC068/NTM1), group IX (AT4G28500/ANAC073/SND2), and group X (AT3G56520). Their bindings to NRS1, NRS2, and MybSt1 were studied using Y1H. The results showed that all these NACs can bind to NRS1, NRS2, and MybSt1 (Figure 6), which suggested that individual proteins belonging to different NAC groups having binding affinity for the same cis-element. This is consistent with the results of other scholars. As an example, Jensen et al. found that nine distantly related NAC TFs were able to bind conserved DNA target sequence with a CGT[GA] core, although with different affinities [45]. Some NACs such as AT3G56520 and AT1G77450 have less binding activity with MybSt1 motif than other NAC members, which may be related to the structure of their conserved NAC domain located at the N-terminal region. The fact that not all genes bind to the same element with the same binding capacity is reasonable, because these genes may be involved in different regulatory networks by preferring to identify different motifs.

All in all, the knowledge of NACs that can bind to MybSt1, NRS1, and NRS2 besides NACRS will contribute to revealing the function of NACs in depth.

## 3. Materials and Methods

### 3.1. TF-Centered Y1H Analysis

A random short DNA sequence insertion library was constructed according to Ji et al. as the prey library [23]. The ORF of ANAC074 was cloned into pGADT7-Rec2 (designated as: pGADT7-ANAC074) at the site between SMART III Sequence and CDS III Sequence using the infusion method (see Supporting information Table S1 for primers). TF-Centered Y1H screening was performed according to Ji et al. [23] to catch the *cis*-regulatory elements bound by ANAC074.

### 3.2. Identification of the Insertion Sequences

The pHIS2 plasmids were rescued from the positive clones identified by Y1H analysis [23,24], and were sequenced. Y1H assay was performed following the protocol of the Matchmaker One-Hybrid System of Clontech (Clontech™, Palo Alta, CA, USA). The insertion sequences together with the left and the right insertion flanking sequences (“GGG” and “CCC”), were analyzed by using PLACE and PlantCare to identify if they were known motifs.

The MybSt1-binding element “GGATA”, ARR1AT motif “NGATT”, and LTRE-1 motif “CCGAAA” were predicted in these insertion sequences. Three tandem copies of “GGATA”, “TGATT”, and “CCGAAA” were respectively cloned into MCS to drive *HIS3* gene in pHIS2. These pHIS2 constructs were respectively co-transformed with pGADT7-ANAC074 into Y187 yeast cells to study their bindings to ANAC074 using Y1H [23,24].

The sequences containing novel DNA motifs were serially deleted to determine the exact DNA sequence recognized by ANAC074. Since the two flanking sequences of the random insertion sequence may also be part of novel DNA motifs, three bases of each flanking sequence together with the insertion sequence, i.e., “CCCTTAGTTTCGGG” and “CCCTGAATCTCGGG”, were used in serial deletion analysis. Three tandem copies of these deletions were cloned into pHIS2, and respectively reacted with ANAC074 using Y1H analysis [23,24]. The sequences of the serial deletions of these two novel motifs are shown as Figure 1.

### 3.3. Assay of ANAC074 Binding to the Two Novel Motifs and the MybSt1 Motif

The two novel motifs were designed as NRS1 and NRS2 (NRS: NAC Recognized Sequence). Three tandem copies of the NRS1 sequence “TAGTT”, NRS2 “GAATC” and the MybSt1 motif sequence “GGATA” together with their respective mutants “GCGTT”, “TAGGG”, “GGGGG” (mutants of NRS1, named NRS1-M1, NRS1-M2 and NRS1-M3, respectively); “TCATC”, “GACGC”, “TCCGA” (mutants of NRS2, named NRS2-M1, NRS2-M2 and NRS2-M3, respectively); “TTATA”, “GTCGA” and “TTCGC” (mutants of MybSt1, named MYB-M1, MYB-M2 and MYB-M3, respectively) were cloned into pHIS2 vector (see Supplemental Table S1 for primers used), respectively. Y1H screening analysis was performed to study their interactions with ANAC074.

To further verify the Y1H result, the DNA-protein interactions were further performed in tobacco leaves. For reporter plasmids construction, three tandem copies of the core sequence of the NRS1 “TAGTT” and its complete mutant “GGGGG”, NRS2 “GAATC” and its complete mutant “TCCGA”, the MybSt1 motif “GGATA” and its complete mutant “TTCGC” were respectively fused to the minimal 35S promoter (−46 to +1) to drive the *GUS* reporter gene (designed as pCAM-NRS1, pCAM-NRS1-M3, pCAM-NRS2, pCAM-NRS2-M3, pCAM-MYB, pCAM-MYB-M3, respectively) (see Supplemental Table S1 for primers used). The effector was constructed by cloning the ORF of ANAC074 into pROKII under 35SCAMV promoter (named as pROKII-ANAC074).

Each of these reporters was co-transformed with effector into tobacco leaves using a particle bombardment method (Bio-Rad, Hercules, CA, USA). Firstly, detached leaves from tobacco (*Nicotiana benthamiana*) were tiled on Murashige & Skoog (MS, PhytoTechnology, Lenexa, KS, USA) agar medium. The procedure of transformation followed the manual for the Biolistic PDS-1000/He Particle Delivery System (Bio-Rad). For coating particles with DNA, 50 μL gold powder suspension with a diameter of 1 μm (60 mg/mL), 5 μL plasmid DNA (1 ug/uL), 50 μL CaCl_2_ (2.5 M), and 20 μL spermidine (0.1 M) was added and then proceeded as follows. Vortex for 10 min, then let sit for 1 min. Spin down briefly (~10 s) to settle loose gold and abandon supernatant. Wash once with 140 μL 70% ethyl alcohol, then spin down briefly. Wash once with 140 μL of 100% ethyl alcohol, then resuspend gold pellet in 48 μL 100% ethyl alcohol. Vortex well before spreading the coated gold out on each flyer. The transformation conditions were 9 cm target distance, 1100 psi helium pressure, and 2 times for the number of bombardments. Transfer to a MS agar medium after bombardment, incubate at 24 °C in the dark for 2 days before examining GUS activity. GUS activity levels were determined according to Jefferson [46]. There were three independent experiments and leaves were taken from 25–30 separate plant individuals per experiment.

### 3.4. Analysis of the Binding of ANAC074 to the Truncated Promoters

The sequences of MybSt1, NRS1, and NRS2 were searched using the Patmatch program on Tair [33] to find the genes that were putatively regulated by ANAC074. The criteria for promoter selection were the promoter regions (0 to −1500 bp) of genes containing fewer kinds of the motifs of MybSt1, NRS1, and NRS2, but having more copies of the contained motif. We found a total of 33 genes that met the criteria. Three of them (*AT4G17500*, *AT1G74930,* and *AT4G11280*) happen to be the target genes of ANAC069 [22]. We speculated that these three genes are probably also the target genes of ANAC074. Therefore, these three genes were selected for the promoter mutagenesis.

The pHIS2 harboring the truncated promoter of *AT4G17500* (without NACRS, NRS2 or MybSt1) containing NRS1 “TAGTT” (pHIS2-NRS1p+), without “TAGTT” (pHIS2-NRS1p-) or with NRS1 complete mutant “GGGGG” (pHIS2-NRS1pm) driving *HIS3* gene, were respectively generated as reporter vectors (see Supplemental Table S1 for primers used). The pHIS2 harboring the truncated promoter of *AT1G74930* (without NACRS, NRS1, or MybSt1) containing NRS2 “GAATC” (pHIS2-NRS2p+), without “GAATC” (pHIS2-NRS2p-) or NRS2 complete mutant “TCCGA” (pHIS2-NRS2pm) driving *HIS3* gene were respectively generated as reporter vectors (see Supplemental Table S1 for primers used). Similarly, the pHIS2 harboring the truncated promoter of *AT4G11280* (without NACRS, NRS1, or NRS2) containing MybSt1 “GGATA” (pHIS2-MYBp+), without “GGATA” (pHIS2-MYBp-) or MybSt1 complete mutant “TTCGC” driving *HIS3* gene were also respectively generated as reporter vectors (see Supplemental Table S1 for primers used). The interactions of these sequences with the ANAC074 were studied using Y1H analysis [23,24].

To further verify the Y1H results, transient expression assays in vivo were performed. The reporter vectors harboring the truncated promoter of *AT4G17500* (same as used in Y1H) containing NRS1 “TAGTT” (pCAM-NRS1p+), without NRS1 (pCAM-NRS1p-) or NRS1 complete mutant “GGGGG” (pCAM-NRS1pm), were respectively generated (see Supplemental Table S1 for primers used). Similarly, the truncated promoter of *AT1G74930* (same as used in Y1H), which contained NRS2 “GAATC” (pCAM-NRS2p+), without “GAATC” (pCAM-NRS2p-) or NRS2 completely mutant “TCCGA” (pCAM-NRS2pm) were respectively fused to the minimal 35S promoter (−46 to +1) to substitute 35S promoter to drive *GUS* as reporter vectors (see Supplemental Table S1 for primers used). The reporter vectors harboring the truncated promoter of *AT4G11280* (same as used in Y1H) containing MybSt1 motif “GGATA” (pCAM-MYBp+), without “GGATA” (pCAM-MYBp-) or MybSt1 complete mutant motif “TTCGC” (pCAM-MYBpm) were also respectively generated. (See Supplemental Table S1 for primers used).

These reporter constructs were respectively co-transformed with effector (pROKII-ANAC074) into tobacco leaves using the particle bombardment method (Bio-Rad, Hercules, CA, USA), respectively. GUS activity levels were determined according to the method of Jefferson [46]. Three independent experiments were performed. The transformation of single reporter plasmid and the empty effector vector were used as a negative control. The empty pCAMBIA1301 was transformed and used as positive control.

### 3.5. ChIP Analysis

To confirm the binding of ANAC074 to NRS1, NRS2, and MybSt1 actually occurs in plants, ChIP analysis was performed. The coding regions of ANAC074 without the termination codon were ligated in frame to the N-terminal of GFP driven by 35SCAMV promoter to generate the ANAC074::GFP fusion gene, and transformed into *Arabidopsis thaliana* in the Columbia ecotype background. The 3-week-old transgenic plants were used for ChIP assay following the procedures of Haring et al. [47]. Briefly, protein and DNA were cross-linked by 3% formaldehyde. The purified cross-linked nuclei were sonicated to shear the chromatin into 0.5–0.7 kb fragments. We saved 1/10 of the volume as input control. The remaining sonicated chromatin was divided into two aliquots. These two aliquots were respectively incubated with GFP antibody (ChIP+) or a rabbit anti-hemagglutinin (HA, Beyotime, Shanghai, China) antibody as a negative control (ChIP-). The antibody-bound complex was precipitated with protein A Agarose beads. The DNA fragments were released from the immunoprecipitated complexes by reversing the cross-linking at 65 °C for 3 h. Immunoprecipitated DNA was purified by chloroform extraction. PCR was performed and visualized by gel electrophoresis. The primers used for PCR are shown in Supplemental Table S1. PCR was performed as follows: 94 °C for 2 min; 40 cycles of 94 °C for 12 s, 58 °C for 30 s, and 72 °C for 30 s; and 72 °C for 5 min.

### 3.6. Comparison of ANAC074 Binding with the Four cis-Acting Elements

To compare the binding activity of ANAC074 to NACRS, MybSt1, NRS1, and NRS2, the effector construct pROKII-ANAC074 was co-transformed with each reporter constructs pCAM-NRS1, pCAM-NRS2, pCAM-MybSt1 or pCAM-NACRS into tobacco leaves using particle bombardment method (Bio-Rad, Hercules, CA, USA). The empty pCAMBIA1301 was transformed and used as positive control. Three independent experiments were performed.

Statistical analyses were carried out using SPSS 16.0 (SPSS Inc., Chicago, IL, USA) software. Data were compared using Student’s t test. Asterisks indicate a significant difference compared with NACRS (* *p* < 0.05).

### 3.7. Analysis of the Interaction of the Other Ten Arabidopsis NAC Family Members with ANAC074-Binding Elements

For effector vectors construction, the ORFs of AT3G44350, AT2G33480, AT3G56520, AT3G04420, AT4G01540, AT2G43000, AT5G50820, AT1G69490, AT1G77450 and AT4G28500 were respectively cloned into pGADT7-Rec2 (designated as: pGADT7-AT3G44350, -AT2G33480, -AT3G56520, -AT3G04420, -AT4G01540, -AT2G43000, -AT5G50820, -AT1G69490, -AT1G77450 and -AT4G28500) at the site between SMART III Sequence and CDS III Sequence using the infusion method. Each of the 10 effector vectors containing NAC gene was co-transformed with reporter vectors pHIS2-NRS1-D4, -D7, -D6, -D8, pHIS2-NRS2-D4, -D5, -D6, -D8, or pHIS2-MybSt1 into yeast cells. The bindings of the 10 NACs to the elements harboring in pHIS2 were analyzed using the Y1H system [23,24].

## 4. Conclusions

In conclusion, we identified the MybSt1-binding element and two novel motifs NRS1 and NRS2, which are bound by NACs, and further showed that ANAC074 may be a regulator functioning upstream of ethylene synthesis genes and stress responsive genes via binding to the MybSt1, NRS1 or NRS2 element. Identifying the cis-acting element recognized by a transcription factor is crucial to parse its function, and the identification of the MybSt1, NRS1, and NRS2 motifs will be beneficial to future NAC gene function analyses.

## Figures and Tables

**Table 1:** Analysis of the insertion sequence that binds to ANAC074.

Clone Number	Random DNA Insertion Sequence (5’–3’) Underlined Together with the Two Sides of Flanking Sequences	Motif Prediction
1	CCCCTTCACGCGGG	Core sequence of NACRS: “CACG”
2	CCCAGGATAACGGG	MybSt1-binding element: “GGATA”
3	CCCTTAGTTTCGGG	LTRE-1: “CCGAAA”
4	CCCTGAATCTCGGG	ARR1AT: “NGATT”

## Supplementary Materials

Supplementary materials can be found at http://www.mdpi.com/1422-0067/19/10/3271/s1.
