# Supplementary material for: Transcription Factor ANAC074 Binds to NRS1, NRS2, or MybSt1 Element in Addition to the NACRS to Regulate Gene Expression
Source: Int J Mol Sci. 2018 Oct 21;19(10):3271. doi: 10.3390/ijms19103271 (PMC6214087; doi:10.3390/ijms19103271)
Supplement: Supplementary file 1 [file ijms-19-03271-s001.pdf]

## Supplemental Table S1

**Sequences of oligonucleotide primers used in this study.**

| Primer names | Primers sequences (5’-3’) |
|---|---|
| pHIS2-NRS1-D0-F | 5’-AATTCCCCTTAGTTTCGGGCCCTTAGTTTCGGGCCCTTAGTTTCGGGGAGCT-3’ |
| pHIS2-NRS1-D0-R | 5’-CCCCGAAACTAAGGGCCCGAAACTAAGGGCCCGAAACTAAGGGG-3’ |
| pHIS2-NRS1-D1-F | 5’-AATTCCCTTAGTTTCGGCCTTAGTTTCGGCCTTAGTTTCGGGAGCT-3’ |
| pHIS2-NRS1-D1-R | 5’-CCCTTAGTTTCGGCCTTAGTTTCGGCCTTAGTTTCGGG-3’ |
| pHIS2-NRS1-D2-F | 5’-AATTCCTTAGTTTCGCTTAGTTTCGCTTAGTTTCGGAGCT-3’ |
| pHIS2-NRS1-D2-R | 5’-CCTTAGTTTCGCTTAGTTTCGCTTAGTTTCGG-3’ |
| pHIS2-NRS1-D3-F | 5’-AATTCTTAGTTTCTTAGTTTCTTAGTTTCGAGCT-3’ |
| pHIS2-NRS1-D3-R | 5’-CGAAACTAAGAAACTAAGAAACTAA-3’ |
| pHIS2-NRS1-D4-F | 5’-AATTCTAGTTTTAGTTTTAGTTTGAGCT-3’ |
| pHIS2-NRS1-D4-R | 5’-CAAACTAAAACTAAAACTAG-3’ |
| pHIS2-NRS1-D5-F | 5’-AATTCAGTTTAGTTTAGTTTGAGCT-3’ |
| pHIS2-NRS1-D5-R | 5’-CAAACTAAACTAAACTG-3’ |
| pHIS2-NRS1-D6-F | 5’-AATTCTAGTTTAGTTTAGTTGAGCT-3’ |
| pHIS2-NRS1-D6-R | 5’-CAACTAAACTAAACTAG-3’ |
| pHIS2-NRS1-D7-F | 5’-AATTCAGTTAGTTAGTTGAGCT-3’ |
| pHIS2-NRS1-D7-R | 5’-CAACTAACTAACTG-3’ |
| pHIS2-NRS1-D8-F | 5’-AATTCTAGTTAGTTAGTGAGCT-3’ |
| pHIS2-NRS1-D8-R | 5’-CACTAACTAACTAG-3’ |
| pHIS2-NRS2-D0-F | 5’-AATTCCCCTGAATCTCGGGCCCTGAATCTCGGGCCCTGAATCTCGGGGAGCT-3’ |
| pHIS2-NRS2-D0-F | 5’-CCCCGAGATTCAGGGCCCGAGATTCAGGGCCCGAGATTCAGGGG-3 |

| | |
|---|---|
| pHIS2-NRS2-D1-F | 5’-AATTCCCTGAATCTCGGCCTGAATCTCGGCCTGAATCTCGGG AGCT-3’ |
| pHIS2-NRS2-D1-R | 5’-CCCGAGATTCAGGCCGAGATTCAGGCCGAGATTCAGGG-3’ |
| pHIS2-NRS2-D2-F | 5’-AATTCCTGAATCTCGCTGAATCTCGCTGAATCTCGGAGCT-3’ |
| pHIS2-NRS2-D2-R | 5’-CCGAGATTCAGCGAGATTCAGCGAGATTCAGG-3’ |
| pHIS2-NRS2-D3-F | 5’-AATTCTGAATCTCTGAATCTCTGAATCTCGAGCT-3’ |
| pHIS2-NRS2-D3-R | 5’-CGAGATTCAGAGATTCAGAGATTCAG-3’ |
| pHIS2-NRS2-D4-F | 5’-AATTCGAATCTGAATCTGAATCTGAGCT-3’ |
| pHIS2-NRS2-D4-R | 5’-CAGATTCAGATTCAGATTCG-3’ |
| pHIS2-NRS2-D5-F | 5’-AATTCAATCAATCAATCGAGCT-3’ |
| pHIS2-NRS2-D5-R | 5’-CGATTGATTGATTG-3’ |
| pHIS2-NRS2-D6-F | 5’-AATTCGAATCGAATCGAATCGAGCT-3’ |
| pHIS2-NRS2-D6-R | 5’-CGATTCGATTCGATTCG-3’ |
| pHIS2-NRS2-D7-F | 5’-AATTCAATCTAATCTAATCTGAGCT-3’ |
| pHIS2-NRS2-D7-R | 5’-CAGATTAGATTAGATTG-3’ |
| pHIS2-NRS2-D8-F | 5’-AATTCGAATGAATGAATGAGCT-3’ |
| pHIS2-NRS2-D8-R | 5’-CATTCATTCATTCG-3’ |
| pHIS2-NRS1-M1-F | 5’-AATTCGCGTTGCGTTGCGTTGAGCT-3’ |
| pHIS2-NRS1-M1-R | 5’-CAACGCAACGCAACGCG-3’ |
| pHIS2-NRS1-M2-F | 5’-AATTCTAGGGTAGGGTAGGGGAGCT-3’ |
| pHIS2-NRS1-M2-F | 5’-CCCCTACCCTACCCTAG-3’ |
| pHIS2-NRS1-M3-F | 5’-AATTCGGGGGGGGGGGGGGGAGCT-3’ |
| pHIS2-NRS1-M3-R | 5’-CCCCCCCCCCCCCCCCG-3’ |
| pHIS2-NRS2-M1-F | 5’-AATTCTCATCTCATCTCATCGAGCT-3’ |
| pHIS2-NRS2-M1-R | 5’-CGATGAGATGAGATGAG-3’ |

| | |
|---|---|
| pHIS2-NRS2-M2-F | 5’-AATTCGACGCGACGCGACGCGAGCT-3’ |
| pHIS2-NRS2-M2-R | 5’-CGCGTCGCGTCGCGTCG-3’ |
| pHIS2-NRS2-M3-F | 5’-AATTCTCCGATCCGATCCGAGAGCT-3’ |
| pHIS2-NRS2-M3-R | 5’-CTCGGATCGGATCGGAG-3’ |
| pHIS2-MYB-F | 5’-AATTCGGATAGGATAGGATAGAGCT-3’ |
| pHIS2-MYB-R | 5’-CTATCCTATCCTATCCG-3’ |
| pHIS2-MYB-M1-F | 5’-AATTCTTATATTATATTATAGAGCT-3’ |
| pHIS2-MYB-M1-R | 5’-CTATAATATAATATAAG-3’ |
| pHIS2-MYB-M2-F | 5’-AATTCGTCGAGTCGAGTCGAGAGCT-3’ |
| pHIS2-MYB-M2-R | 5’-CTCGACTCGACTCGACG-3’ |
| pHIS2-MYB-M3-F | 5’-AATTCTTCGCTTCGCTTCGCGAGCT-3’ |
| pHIS2-MYB-M3-R | 5’-CGCGAAGCGAAGCGAAG-3’ |
| pCAM-NRS1-F | 5’-AGCTTTAGTTTAGTTTAGTTACCCTTCCTCTATATAAGGAAGT TCATTTCATTTGGAGAGAACACGGC-3’ |
| pCAM-NRS1-R | 5’-CATGGCCGTGTTCTCTCCAAATGAAATGAACTTCCTTATATAG AGGAAGGGTAACTAAACTAAACTAA-3’ |
| pCAM-NRS1-M3-F | 5’-AGCTTGCTGGGCTGGGCTGGACCCTTCCTCTATATAAGGAAG TTCATTTCATTTGGAGAGAACACGGC-3’ |
| pCAM-NRS1-M3-R | 5’-CATGGCCGTGTTCTCTCCAAATGAAATGAACTTCCTTATATAG AGGAAGGGTCCAGCCCAGCCCAGC-A-3’ |
| pCAM-NRS2-F | 5’-AGCTTGAATCGAATCGAATCACCCTTCCTCTATATAAGGAAGT TCATTTCATTTGGAGAGAACACGGC-3’ |
| pCAM-NRS2-R | 5’-CATGGCCGTGTTCTCTCCAAATGAAATGAACTTCCTTATATAG AGGAAGGGT-GATTCGATTCGATTCA-3’ |
| pCAM-NRS2-M3-F | 5’-AGCTTTCCGATCCGATCCGAACCCTTCCTCTATATAAGGAAGT TCATTTCATTTGGAGAGAACACGGC-3’ |
| pCAM-NRS2-M3-R | 5’-CATGGCCGTGTTCTCTCCAAATGAAATGAACTTCCTTATATAG AGGAAGGGT-TCGGATCGGATCGGAA-3’ |
| pCAM-MYB-F | 5’-AGCTTGGATAGGATAGGATAACCCTTCCTCTATATAAGGAAGT TCATTTCATTTGGAGAGAACACGGC-3’ |
| pCAM-MYB-R | 5’-CATGGCCGTGTTCTCTCCAAATGAAATGAACTTCCTTATATAG AGGAAGGGT-TATCCTATCCTATCCA-3’ |
| pCAM-MYB-M3-F | 5’-AGCTTTTCGCTTCGCTTCGCACCCTTCCTCTATATAAGGAAGT TCATTTCATTTGGAGAGAACACGGC-3’ |

| | |
|---|---|
| pCAM-MYB-M3-R | 5’-CATGGCCGTGTTCTCTCCAAATGAAATGAACTTCCTTATATAG AGGAAGGGTGCGAAGCGAAGCGAAA-3’ |
| pROK-ANAC074-F | 5’-CACGGGTACCATGGGTTTGAAAGATATTGG-3’ |
| pROK-ANAC074-R | 5’-CCCGAGCTCTCATTGGAAAGCGAGGATATTTTC-3’ |
| pHIS2-NRS1p+-F | 5’-ATCGGAATTCTAGTTTAGCTCACATGACGGGG-3’ |
| pHIS2-NRS1p+-R | 5’-ACCGAGCTCAACTACAAATATATATTATGAC-3’ |
| pHIS2-NRS1p--F | 5’-ATCGGAATTCTAGCTCACATGACGGGGTTTTT-3’ |
| pHIS2-NRS1p--R | 5’-ACCGAGCTCCAAATATATATTATGACTCCTTA-3’ |
| pHIS2-NRS1pm-F | 5’-ATCGGAATTCGCTGGTAGCTCACATGACGGGGTTTTT-3’ |
| pHIS2-NRS1pm-R | 5’-ACCGAGCTCCCAGCCAAATATATATTATGACTCCTTA-3’ |
| pHIS2-NRS2p+-F | 5’-ATCGGAATTCGAATCTAAGGGAGACTGTTCTTAC-3’ |
| pHIS2-NRS2p+-R | 5’-ACCGAGCTCGATTCGTAGAAGAAGAAAGGTG-3’ |
| pHIS2-NRS2p--F | 5’-ATCGGAATTCTAAGGGAGACTGTTCTTACACAAG-3’ |
| pHIS2-NRS2p--R | 5’-ACCGAGCTCGTAGAAGAAGAAAGGTGGAACC-3’ |
| pHIS2-NRS2pm-F | 5’-ATCGGAATTCTCCGAAAGGGAGACTGTTCTTAC-3 |
| pHIS2-NRS2pm-R | 5’-ACCGAGCTCTCGGAGTAGAAGAAGAAAGGTG-3’ |
| pHIS2-MYBp+-F | 5’-ATCGGAATTCGGATATTTTTTTTTGTCATCGTG-3’ |
| pHIS2-MYBp+-R | 5’-ACCGAGCTCTATCCGTTCAAATTATAGAACATG-3’ |
| pHIS2-MYBp--F | 5’-ATCGGAATTCTTTTTTTTTGTCATCGTGACGATA-3’ |
| pHIS2-MYBp--R | 5’-ACCGAGCTCGTTCAAATTATAGAACATGCG-3’ |
| pHIS2-MYBpm-F | 5’-ATCGGAATTCTTCGCTTTTTTTTTGTCATCGTG-3’ |
| pHIS2-MYBpm-R | 5’-ACCGAGCTCGCGAAGTTCAAATTATAGAACATG-3’ |
| pCAM-NRS1p+-F | 5’-ATCGAAGCTTAGTTTAGCTCACATGACGGGG-3’ |
| pCAM-NRS1p+-R | 5’-ACCCCATGGCCGTGTTCTCTCCAAATGAAATGAACTTCCTTAT ATAGAGGAAGGGTAACTACAAATATATATTATGAC-3’ |
| pCAM-NRS1p--F | 5’-ATCGAAGCTTTAGCTCACATGACGGGGTTTTTTT-3’ |

| | |
|---|---|
| pCAM-NRS1p--R | 5’-ACCCCATGGCCGTGTTCTCTCCAAATGAAATGAACTTCCTTAT ATAGAGGAAGGGTCAAATATATATTATGACTCCTTAATG-3’ |
| pCAM-NRS1pm-F | 5’-ATCGAAGCTTGCTGGTAGCTCACATGACGGGGTTTTTTT-3’ |
| pCAM-NRS1pm-R | 5’-ACCCCATGGCCGTGTTCTCTCCAAATGAAATGAACTTCCTTAT ATAGAGGAAGGGTCCAGCCAAATATATATTATGACTCCTTAATG -3’ |
| pCAM-NRS2p+-F | 5’-ATCGAAGCTTGAATCTAAGGGAGACTGTTCTTAC-3’ |
| pCAM-NRS2p+-R | 5’-ACCCCATGGCCGTGTTCTCTCCAAATGAAATGAACTTCCTTAT ATAGAGGAAGGGTGATTCGTAGAAGAAGAAAGGTG-3’ |
| pCAM-NRS2p--F | 5’-ATCGAAGCTTTAAGGGAGACTGTTCTTACACAAG-3’ |
| pCAM-NRS2p--R | 5’-ACCCCATGGCCGTGTTCTCTCCAAATGAAATGAACTTCCTTAT ATAGAGGAAGGGTGTAGAAGAAGAAAGGTGGAACC-3’ |
| pCAM-NRS2pm-F | 5’-ATCGAAGCTTTCCGATAAGGGAGACTGTTCTTAC-3’ |
| pCAM-NRS2pm-R | 5’-ACCCCATGGCCGTGTTCTCTCCAAATGAAATGAACTTCCTTAT ATAGAGGAAGGGTTCGGAGTAGAAGAAGAAAGGTGGAACCA -3’ |
| pCAM-MYBp+-F | 5’-ATCGAAGCTTGGATATTTTTTTTTGTCATCGTG-3’ |
| pCAM-MYBp+-R | 5’-ACCCCATGGCCGTGTTCTCTCCAAATGAAATGAACTTCCTTAT ATAGAGGAAGGGTTATCCGTTCAAATTATAGAACATG-3’ |
| pCAM-MYBp--F | 5’-ATCGAAGCTTTTTTTTTTTGTCATCGTGACGATA-3’ |
| pCAM-MYBp--R | 5’-ACCCCATGGCCGTGTTCTCTCCAAATGAAATGAACTTCCTTAT ATAGAGGAAGGGTGTTCAAATTATAGAACATGCGA-3’ |
| pCAM-MYBpm-F | 5’-ATCGAAGCTTTTCGCTTTTTTTTTGTCATCGTGACG-3’ |
| pCAM-MYBpm-R | 5’-ACCCCATGGCCGTGTTCTCTCCAAATGAAATGAACTTCCTTAT ATAGAGGAAGGGTGCGAAGTTCAAATTATAGAACATG-3’ |
| pCAM-NACRS-F | 5’-AGCTTTCTTCTGTAACACGCATGTGACCCTTCCTCTATATAAG GAAGTTCATTTCATTTGGAGAGAACACGGC-3’ |
| pCAM-NACRS-R | 5’-CATGGCCGTGTTCTCTCCAAATGAAATGAACTTCCTTATATAG AGGAAGGGTCACATGCGTGTTACAGAAGAA-3’ |
| ChIP-AT2G21320-F1 | 5’-TCAGCTTAGCAAAAGTACAC-3’ |
| ChIP-AT2G21320-R1 | 5’-ACATTTTTAGGAATATGTT-3’ |
| ChIP-AT2G21320-F2 | 5’-GAGAGATTTAGTGAGAGCTT-3’ |
| ChIP-AT2G21320-R2 | 5’-GCAGTGTCAAAAATGGTCTC-3’ |
| ChIP-AT2G21320-F3 | 5’-CTGGGTTAACATTTGCTTG-3’ |

| | |
|---|---|
| ChIP-AT2G21320-R3 | 5’-CTGAAGTATCAATTGGAGC-3’ |
| ChIP-AT2G31880-F1 | 5’-GTACAATTTAGCCCATTTCAC-3’ |
| ChIP-AT2G31880-R1 | 5’-ACTGAATTCTTACATAAGAT-3’ |
| ChIP-AT2G31880-F2 | 5’-CATCCTCGTTAGCTATCTGG-3’ |
| ChIP-AT2G31880-R2 | 5’-GTTCCATAGTGAGCCACCG-3’ |
| ChIP-AT2G31880-F3 | 5’-CCGGTTTTGTAGAACCGGG-3’ |
| ChIP-AT2G31880-R3 | 5’-GTTATGTTACACACACCTTC-3’ |
| ChIP-AT3G13470-F1 | 5’-CTAAAAGCAAATCATTAAG-3’ |
| ChIP-AT3G13470-R1 | 5’-TTAGAACTCTCTAGAACGT-3’ |
| ChIP-AT3G13470-F2 | 5’-TTTCTTCTATAAATTTACC-3’ |
| ChIP-AT3G13470-R2 | 5’-CGATCTTAAAATGTTTTTAT-3’ |
| ChIP-AT3G13470-F3 | 5’-GAAGACTATAGACCGTGTG-3’ |
| ChIP-AT3G13470-R3 | 5’-TTTTTAAAGATATTTCAATG-3’ |
| pGAD-ANAC074-F | 5’-CAGAGTGGCCATTATGGCCC-ATGGGTTTGAAAGATATTG-3’ |
| pGAD-ANAC074-R | 5’-GAGGCCGAGGCGGCCGACATG-TCATTGGAAAGCGAGGATA-3’ |
| pGAD-AT3G44350-F | 5’-CAGAGTGGCCATTATGGCCC-ATGGGAGAAGAGCTTTCTG-3’ |
| pGAD-AT3G44350-R | 5’-GAGGCCGAGGCGGCCGACATG-TTAAGACCAATTCATTTG-3’ |
| pGAD-AT2G33480-F | 5’-CAGAGTGGCCATTATGGCCC-ATGGAGAAGAGGAGCTC-3’ |
| pGAD-AT2G33480-R | 5’-GAGGCCGAGGCGGCCGACATG-CTATAGAAACAAACAAAAC-3’ |
| pGAD-AT3G56520-F | 5’-CAGAGTGGCCATTATGGCCC-ATGGAGCCAACACACAG-3’ |
| pGAD-AT3G56520-R | 5’-GAGGCCGAGGCGGCCGACATG-CTATTCGGATTCGGAATC-3’ |
| pGAD-AT3G04420-F | 5’-CAGAGTGGCCATTATGGCCC-ATGGAGAATCCGGTGGG-3’ |
| pGAD-AT3G04420-R | 5’-GAGGCCGAGGCGGCCGACATG-TTATGTTCTTGAGATAGAAG-3’ |
| pGAD-AT4G01540-F | 5’-CAGAGTGGCCATTATGGCCC-ATGATGAAAGGTCTGATTGG-3’ |

| | |
|---|---|
| pGAD-AT4G01540-R | 5’-GAGGCCGAGGCGGCCGACATG-TCATAACTTCTTCACCGG-3’ |
| pGAD-AT2G43000-F | 5’-CAGAGTGGCCATTATGGCCC-ATGAGTGGCGAAGGTAAC-3’ |
| pGAD-AT2G43000-R | 5’-GAGGCCGAGGCGGCCGACATG-CTAGGGTTTAGTGTTGCC-3’ |
| pGAD-AT5G50820-F | 5’-CAGAGTGGCCATTATGGCCC-ATGGACATTTCTGCGCAG-3’ |
| pGAD-AT5G50820-R | 5’-GAGGCCGAGGCGGCCGACATG-TCACCGGCTTTGAATAAC-3’ |
| pGAD-AT1G69490-F | 5’-CAGAGTGGCCATTATGGCCC-ATGGAAGTAACTTCCCAATC-3’ |
| pGAD-AT1G69490-R | 5’-GAGGCCGAGGCGGCCGACATG-CTAAAACTTAAACATCGCTTG-3’ |
| pGAD-AT1G77450-F | 5’-CAGAGTGGCCATTATGGCCC-ATGATGAAATCTGGGGCTG-3’ |
| pGAD-AT1G77450-R | 5’-GAGGCCGAGGCGGCCGACATG-TCAGAAAGTTCCCTGCC-3’ |
| pGAD-AT4G28500-F | 5’-CAGAGTGGCCATTATGGCCC-ATGACTTGGTGCAATGAC-3’ |
| pGAD-AT4G28500-R | 5’-GAGGCCGAGGCGGCCGACATG-TTAAGGGATAAAAGGTTG-3’ |
